# Annular multi-focal-phase mask multiplexing based large depth of field imaging by interferenceless coded aperture correlation holography

**DOI:** 10.1038/s41598-023-37651-7

**Published:** 2023-07-18

**Authors:** Chao Liu, Yuhong Wan, Teng Ma, Tian Ma, Tianlong Man

**Affiliations:** 1 School of Physics and Optoelectronics, Faculty of Science, Beijing University of Technology, 100 Ping Le Yuan, Chao yang district, Beijing, 100124 China; 2 System Engineering Institute, Academy of Military Science, Beijing, 100010 China

**Keywords:** Imaging and sensing, Imaging techniques

## Abstract

Extending depth-of-field (DOF) of the imaging system without modifying the structure and sacrificing imaging performances of the optical system is of great significance to broaden the capability and application of the imaging system. In this paper, the interferenceless coded aperture correlation holography(I-COACH) is developed to be a large-depth incoherent imaging system by employing an annular multi-focal coded phase mask (AM-CPM). Based on the analyses of axial defocus characteristics in I-COACH, the defocus compensation function is defined, the AM-CPM is designed and multiplexed on the system optical pupil, which plays the role of a gradual lens. In AM-CPM, multi-annular zones with different focal lengths are used to compensate different axial defocus aberrations and adjacent annular zones have symmetric axial defocus aberration correction capability according to the imaging characteristics of the system. The simulations and experimental results fully demonstrate that the axial point spread function distribution of the system obtained by AM-CPM is continuous and the development method enables the extension of the DOF of the I-COACH system by only single exposure point spread hologram. This solution is expected to provide great potential in the field of microscopic imaging and other fields of that based on I-COACH system.

## Introduction

Optical imaging technologies provide the most intuitive and effective way to observe the morphology, structure and even functional information of the samples. Large depth imaging can offer the whole information of expected volume once, thus both large enough imaging depth of field and high spatial resolution are pursued for imaging the structure of a whole samples at the same time or 3D single particle tracking in thick samples. Various methods of depth-of-field (DOF) extension have been developed in imaging systems, such as wave-front coding technology^1–3^, image fusion methods^4^, multi-plane imaging based on digital holography^5^ and scattering based methods^6^, and DOF engineering^7^. Compared to other DOF extension imaging methods with complex matching algorithms and expensive diffractive optics, wave-front encoding technique achieves large DOF imaging by placing a phase mask on the optical pupil of the conventional imaging system to encode and modulate light waves, making the point spread function and optical transfer function of the system are not sensitive to the defocus aberration over the DOF range^8^. Therefore, wave-front encoding enables DOF extension without changing any structure of the imaging system, thus enhancing the system imaging performance.

As one of the incoherent 3D imaging techniques, interferenceless coded aperture correlation holography (I-COACH) technique attracted the focus of increasing research interest in recent years for its advantages such as wide spectrum, non-scanning 3D imaging and higher axial resolution compare to Fresnel incoherent correlation holography^9–11^, and various imaging enhancement methods^12–19^ and potential application sceneries have been development^20–25^. This technique modulates the incident light waves by a wave-front pseudo-random coded phase mask (CPM) to record a series of point spread holograms (PSHs) along z-axial depth and one object hologram (OH) and enables thick 3D objects to be imaged within a calibrated axial range. Based on the imaging scheme of the I-COACH, it is also advantageous to combine wave-front encoding technique for modifying I-COACH as one of incoherent large depth imaging technique. The axial imaging properties of I-COACH system were proposed for the first time in the literature^8^, and the radial quadratic phase function (RQPF) was introduced to implement depth of focus engineering (DOFE). In DOFE, RQPF is enabled to image different depths of discontinuous sub-volume lengths by different modulation parameters.

Different from DOEF, in this paper, to the best of our knowledge, we first analyses the source of axial defocus aberrations for any axial point-object of the I-COACH system, and clarify the axial defocus characteristics and defined the defocus compensation function. Based on the defocus compensation function, we designed an annular multi-focal coded phase mask that is multiplexed on the imaging system pupil plane to compensate simultaneously the defocus aberration with z-depth varies. The proposed method of annular multi-focal coded phase mask multiplexing is coined as AM-CPM, which enables the extension of the DOF of the I-COACH system by only single exposure PSH. The axial point spread function (PSF) distribution of the system obtained by AM-CPM is continuous, and the corresponding parameters can be set according to the sample thickness to adjust the depth of field of the system. The proposed method has significant potential to further extend the application fields of I-COACH such as large DOF microscopic imaging.

## Results

### The mutual constraint analysis of parameters m and α

According to Eqs. (5a) and (5b), the AM-CPM adjacent different ring zones have axially symmetric defocused aberration quadratic phase functions, which play the role of a gradual lens to extend the imaging DOF. Therefore, by modulating the parameters* m* and* α*, it is possible to obtain multifocal encoded phase masks with different DOF extensions.

**Figure 1:**
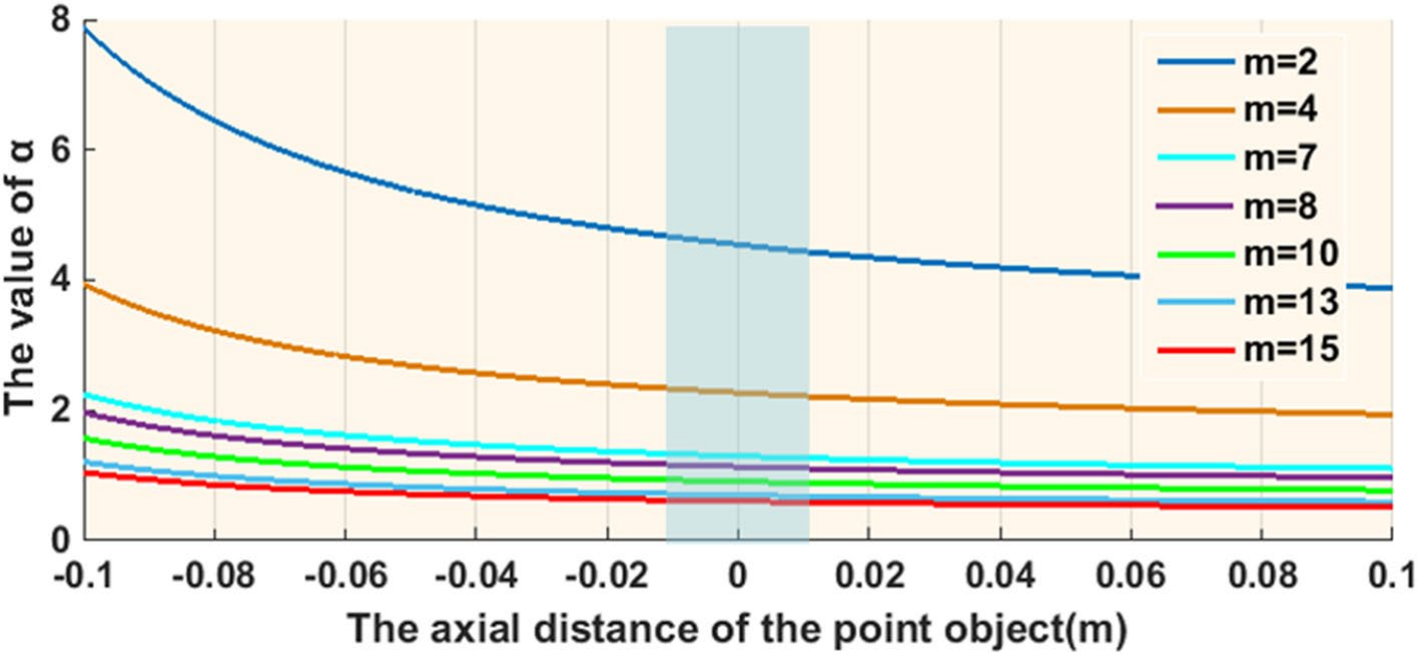
Curves of* d*_*0*_ related to *m* and *α* for − 0.1 m ≤ *d*_*0*_ ≤ 0.1 m.

**Figure 2:**
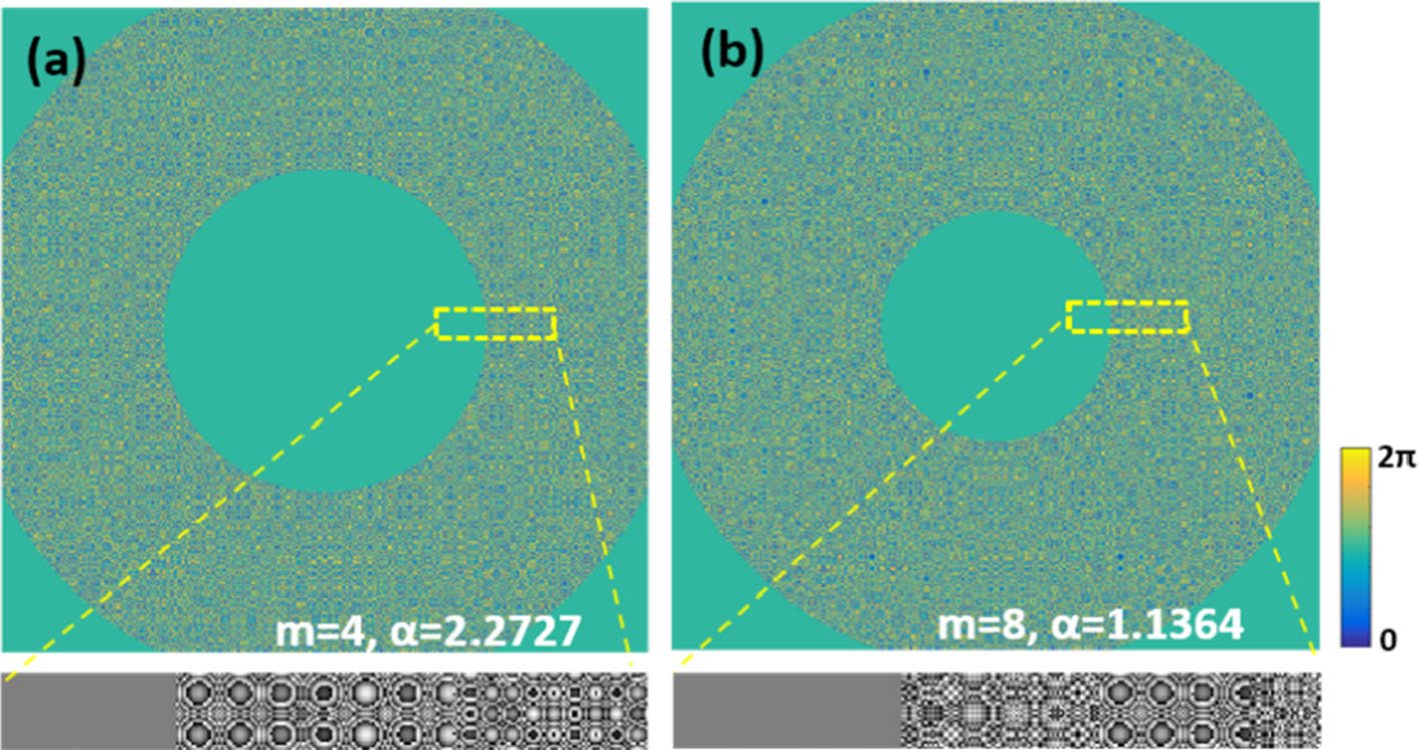
AM-CPM with different modulation parameters *m* and *α*.

In order to enable the system response of the object point in the CCD plane at any axial position to satisfy the conjugate relationship, it is necessary to calculate the correction of the system axial position* d*_*0*_ with respect to the parameters *m* and *α*. As shown in Fig. 1, the front focal plane of the input lens L_0_ is defined as the coordinate origin, with the positive direction to the left and the negative direction to the opposite. According to Eq. (4), the *d*_*0*_ and *α* curve are calculated for the axial range − 0.1 m ≤ *d*_*0*_ ≤ 0.1 m when *m* is taken as 2,4,7,8,10,13 and 15, respectively, and the results are shown in Fig. 1. According to the Fig. 1, d0 is a non-linear relationship with parameter *α* when* m* is a certain determined value. Therefore, the imaging of the two sides of the input lens L_0_ in the I-COACH system is a non-symmetrical relationship, and in the same axial range, the axial defocus aberration of the lens L_0_ in the negative direction is greater than that in the positive direction, which leads to different axial resolution of the two sides of the lens L_0_ in the system. Meanwhile, when *m* is fixed at a certain value, different axial positions correspond to different values of *α*. However, the spatial light modulator (SLM) is loaded with a phase mask that can only satisfy the *α* value at a certain axial position. As a result, the object points in other axial positions are not imaged due to the non-linear relationship, which makes the object points quickly defocus when imaging in the CCD plane. Furthermore, as shown in the blue rectangular wireframe in Fig. 1 that *α* values approximately satisfy a certain linear relationship for a larger choice of *m* in a smaller axial range. The larger the *m* value, the smaller the fluctuation range of *α* values. Therefore, in the I-COACH system, the larger the axial modulation range corresponding to a certain *α* value is chosen, the larger the DOF ranges of the corresponding system. However, with the increasing of* m*, the effective area of each defocus compensation ring on the AM-CPM decreases. As the results, the equivalent numerical aperture of the system decreases, which causes the system to lose a certain degree of transverse resolution. At the same time, another important parameter (one of the very key points in the I-COACH-like systems), the signal to noise (SNR) ratio of the images on each foci within the extended DOF decreases, as well. With the increasing of *m*, the SNR of the in-focused images on each axial plane within the extended DOF gradually decreases. In this paper, the SNRs of the reconstructed images with *m* > 10 were defined to be unacceptable in the following experiments. Therefore, *m* = 10 is chosen as the upper limit of the extension of the DOF in the proposed method. Taking *m* = 4 and *m* = 8 as examples, the AM-CPM is calculated as shown in Fig. 2a and b.

### Simulation validation

**Figure 3:**
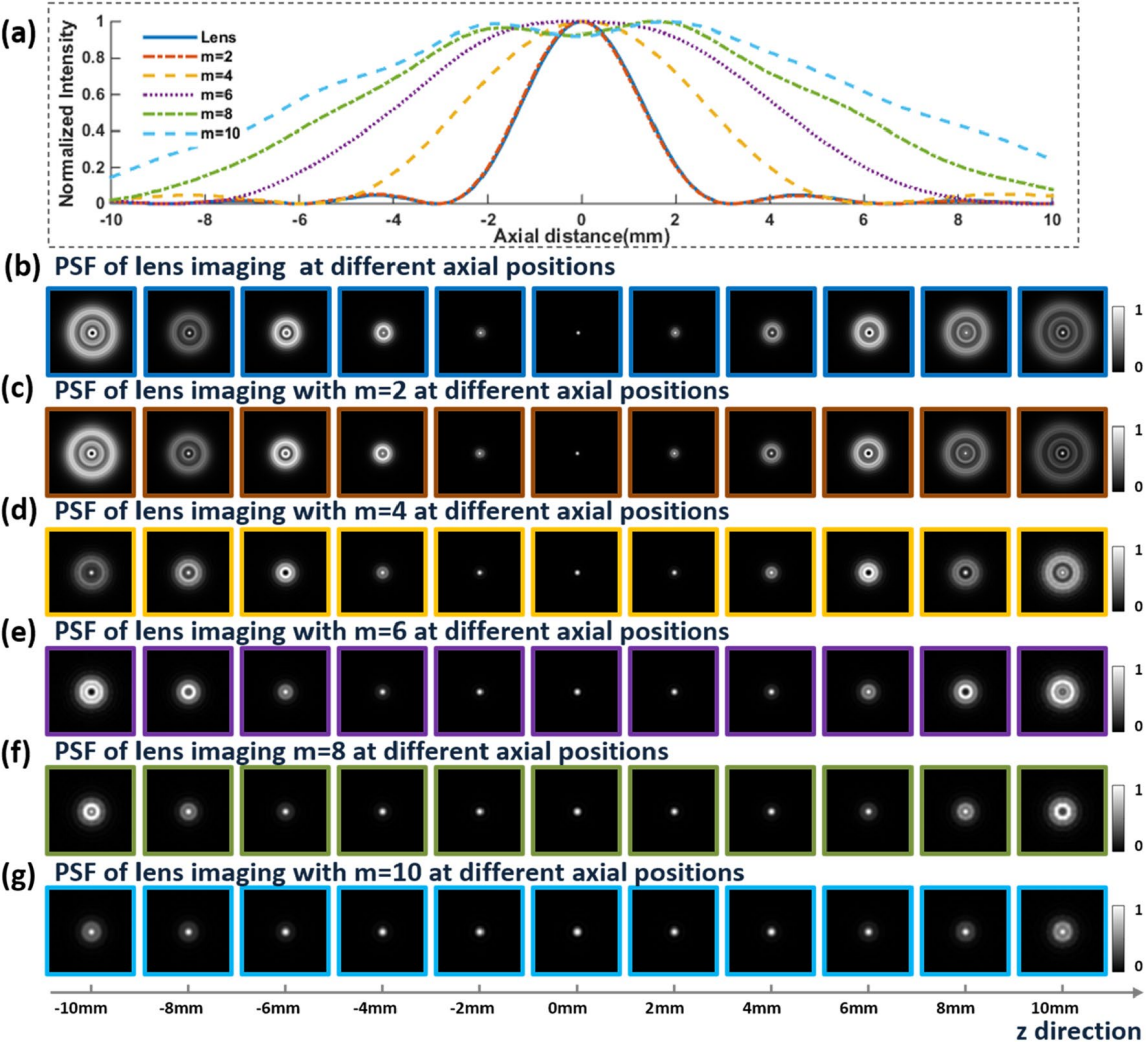
The center curves of axial PSF obtained from lens imaging and the PSF of the point object at different axial positions with different *m*. (**a**)the center curves of axial PSF with different* m*; (**b**) the PSF of the point object under the lens imaging at different axial positions. (**c**–**g**) the PSF of the point object with different *m* under the lens imaging at different axial positions.

**Figure 4:**
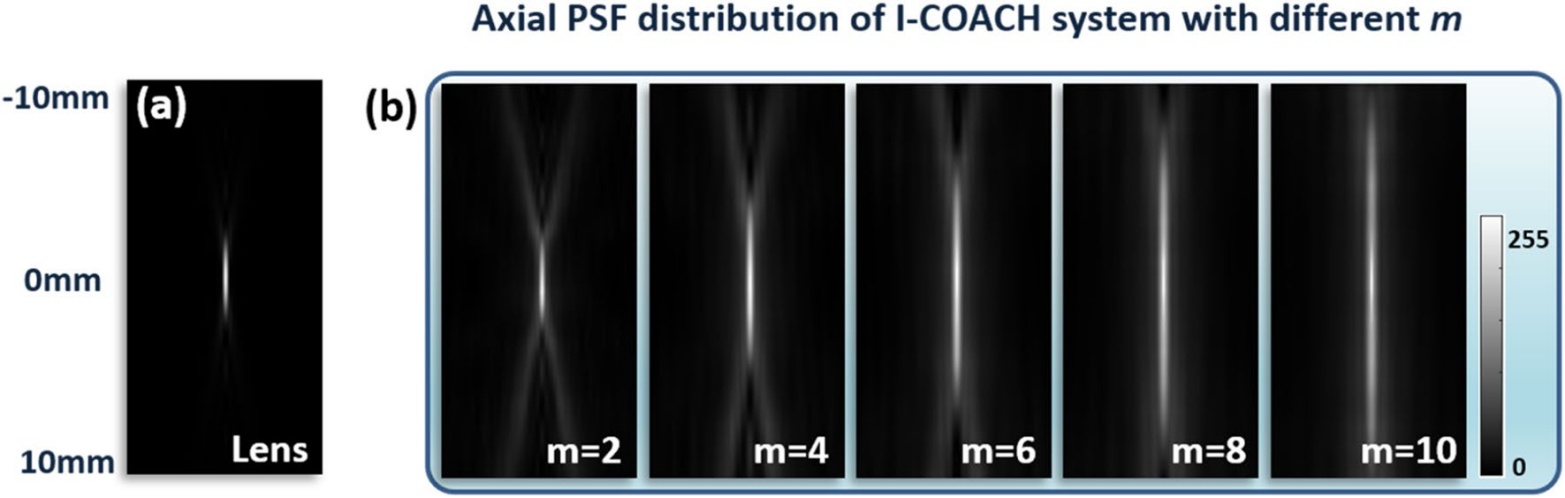
The distribution of the axial PSF of the point object corresponding to different *m*. (**a**) axial PSF distribution of lens imaging; (**b**) axial PSF distribution of point object of the I-COACH system with non-linear reconstruction parameters *p* =  − 0.3 and* o* = 0.8.

Based on the optical setup of the regular I-COACH system, the I-COACH systems have similar transverse and axial resolution as lens-based imaging systems, therefore, switching between the imaging modes of the regular lens imaging and the I-COACH system can be achieved by changing the form of the mask loaded by SLM. Hence, the theory and the effectiveness of AM-CPM for DOF extension of the lens imaging system were verified by simulation firstly. According to the experimental system corresponding to Fig. 8, the simulation was implemented by MATLAB software with the simulation parameters set as λ = 625 nm, and *f*_L0_ = *f*_slm_ = *d*_*s*_ = 150 mm, *d*_*i*_ = 55 mm, Δ*x* = Δ*y* = 8 μm, *M* = *N* = 1080pixel, the aperture ***A*** radius of 3 mm, and the point object diameter of 8 μm. Simulation of the axial PSF distribution of lens imaging in the range − 10 mm ≤ *d*_*0*_ ≤ 10 mm for point objects with values of 2, 4, 6, 8 and 10 for *m*, respectively. The center curves of axial PSF obtained from lens imaging and the PSF of the point object under the lens imaging at different axial positions with different *m* are shown in Fig. 4. As shown in Fig. 3a, the full width at half maxima (FWHM) of the axial direction PSF at 0 mm shows that the PSF is distributed asymmetrically, with lower axial resolution in the positive direction than in the negative direction, which is consistent with the above theoretical analysis that I-COACH has similar imaging properties to regular lens imaging^10^. The axial imaging resolution of the proposed method, for *m* = 10 for example, can be quantified using the FWHM of the corresponding curve in the Fig. 3a. The PSF normalized intensities of point objects at different axial positions are shown in Fig. 3c–g, and it can be clearly seen that a larger *m* corresponding to an AM-CPM with a better DOF extension capability.

Further, we verify the DOF extension capability of AM-CPM in the I-COACH system by simulations. The simulation parameters are the same as above, with the SLM loaded synthetic coded phase mask (SCPM) synthesized from CPM, AM-CPM and QPM with a focal length *f*_slm_ = 150 mm, where the annular sparse CPM is obtained by the modified GS algorithms and the scattering degree σ = 0.167^16–18^. The PSHs of single exposures at different axial positions were obtained, and the non-linear reconstruction (NLR)^15^ was used to obtain the I-COACH axial PSF distribution corresponding to different *m* in the range of − 10 mm ≤ *d*_*0*_ ≤ 10 mm for point objects. The distribution of the axial PSF of the point object corresponding to different *m* is shown in Fig. 4. The PSF obtained by SCPM has a continuous distribution over a certain range.

### The experiment of DOF extension in I-COACH system

**Figure 5:**
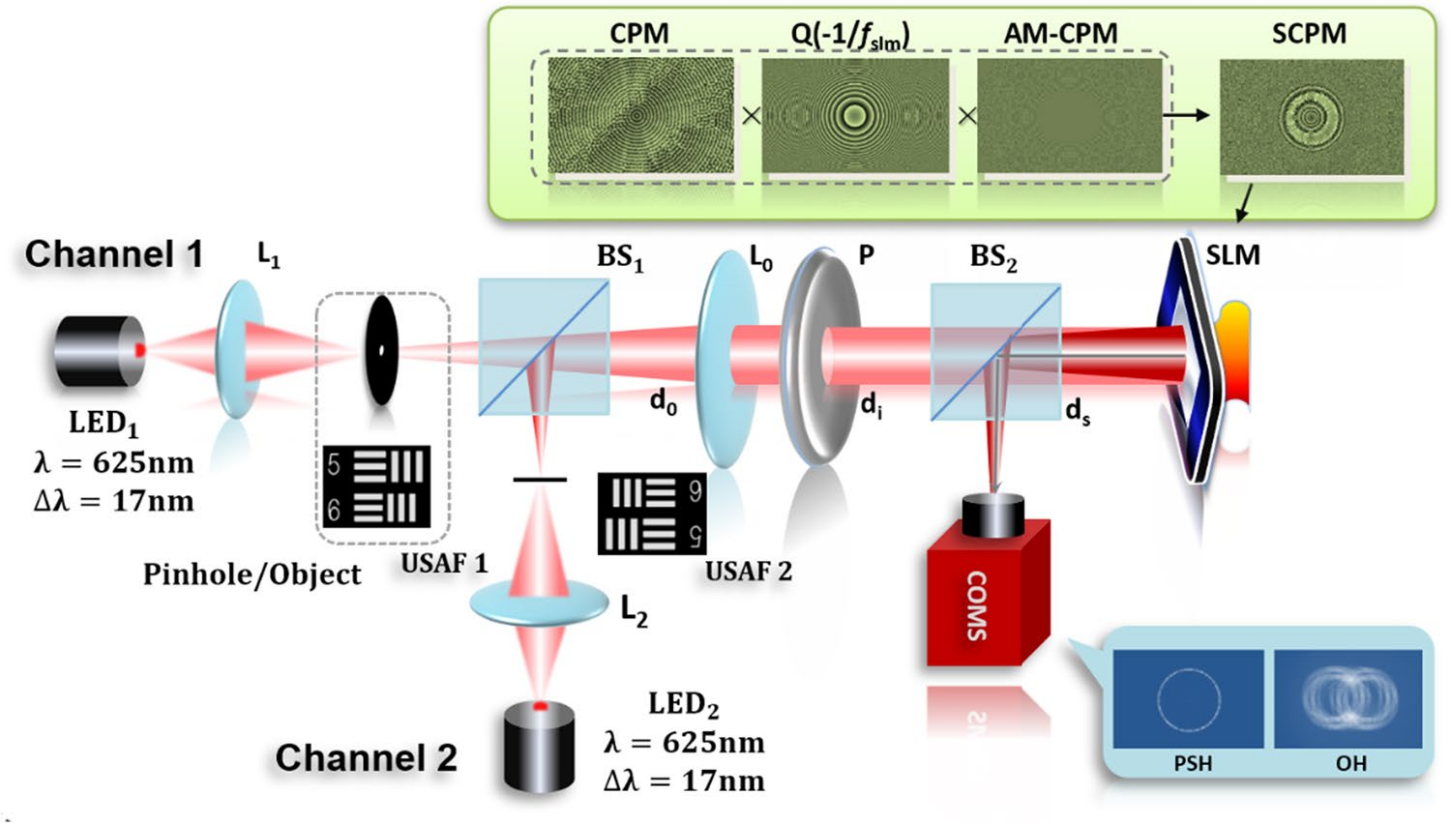
Experimental setup of the I-COACH system.

As shown in Fig. 5, a two-channel experimental system was setup to verify the DOF extension capability of the proposed AM-CPM for the I-COACH system. In experiments, a pinhole or object was illuminated by the incoherent light emitting diode (LED) (Thorlabs LED 625L4, 700mW, center wavelength of λ = 625 nm, Δλ = 17 nm), and the light from beam splitter BS_1_ was collimated by the lens L_0_ and passed through a polarizer** P**. The polarizer **P** polarizes the light along the orientation of the active axis of the spatial light modulator (SLM, Holoeye PLUTO, 1080 × 1920 pixels, 8 μm pixel pitch, phase-only modulation) located at a distance of 55 mm from the L_0_. On the SLM, a SCPM phase mask is displayed whereas its phase is the combination of CPM with the scattering degree σ = 0.167, AM-CPM and QPM. The light modulated by the SLM was collected by a Charge Coupled Device (CCD, Thorlabs CS235MU, 1200 × 1920pixels, 5.86 μm pixel pitch, and monochrome) located at a distance of *d*_*s*_ = 171 mm from the SLM.

**Figure 6:**
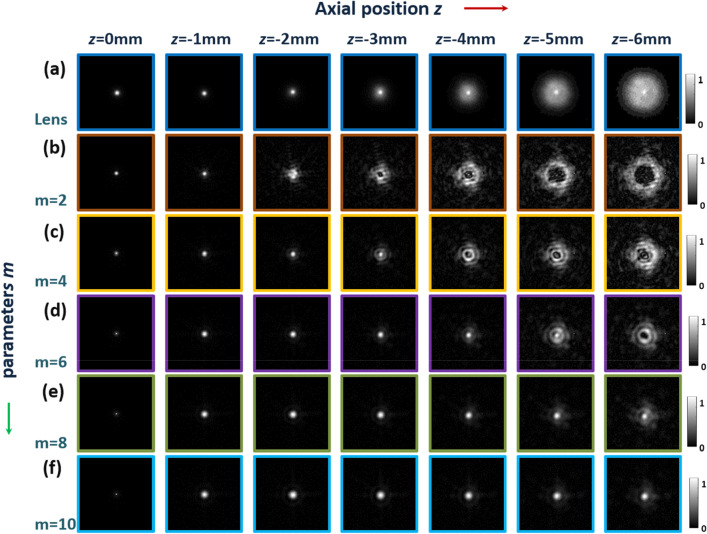
Comparison of PSFs obtained from 20 μm pinhole lens imaging and I-COACH imaging with different *m*.

Corresponding to the simulation results in Fig. 4, in the first experiments, only channel 1 worked and the AM-CPM corresponding to the different *m* was used to record the PSHs. Taking *m* = 2 as an example, the SCPM corresponding to *m* = 2 was loaded into the SLM and the PSHs of 20 μm pinhole at different axial positions were recorded in steps of 1 mm within − 6 mm ≤ *d*_*0*_ ≤ 0 mm. The PSH recorded at the axial position of 0 mm was separately reconstructed with the PSH at other axial positions using an NLR method with a modulation parameter of *p* = − 0.3 and *o* = 0.8 to obtain pinhole reconstructed images to demonstrate the DOF extension capability of the AM-CPM for the I-COACH system. The corresponding PSHs were obtained in the similar way for different *m* and the normalized reconstructed image of the pinhole was obtained from the NLR, the results of which are shown in Fig. 6. Comparison with the lens imaging in Fig. 6a shows that different *m* has different DOF extension range and can obtain a better pinhole reconstruction image within the effective DOF modulation range. Corresponding to Fig. 3, when *m* is within the relative FWHM, well reconstructed images are obtained for different axial point objects, and when the axial distance of the point object passes the maximum modulation depth of the corresponding *m*, the reconstructed image of the point object is fast defocused.

**Figure 7:**
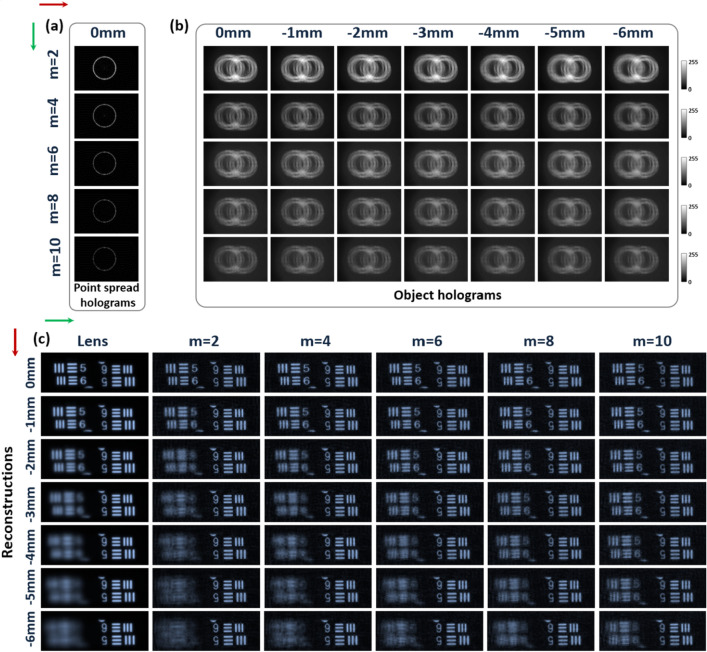
Hologram and non-linear reconstruction images. (**a**,**b**) two-channel I-COACH system holograms of the pinhole with 20 μm and objects at different depth planes corresponding to different *m*; (**c**) The non-linear reconstructed image of the hologram corresponding to Fig. 8b.

Furthermore, two United States Air Force (USAF (GO Edmund optics, USAF 1951 1X and Newport, USAF 1951 RES-1)) targets placed on the front focal plane of input lens L_0_ of channel 1 and channel 2 respectively, and the USAF target group of 3 was illuminated. Taking *m* = 2 as an example, the SCPM corresponding to* m* = 2 is loaded into the SLM, fixing the channel 2 object at the 0 mm position and moving the channel 1 object within − 6 mm ≤ *d*_*0*_ ≤ 0 mm in steps of 1 mm to record the object hologram OHs of the two-channel object at different axial positions, and the holograms of the PSHs and OHs are shown in Fig. 7a and b. The single exposure PSH of the pinhole at the 0 mm position was obtained separately from the OHs at different axial positions using NLR method with a modulation parameter of *p* = − 0.3 and *o* = 0.8 to obtain a reconstructed image. The corresponding OHs and object reconstruction images were obtained in the same way for different *m*. The results are shown in Fig. 7c. It can be seen that different *m* can achieve simultaneous imaging of objects at different depth planes in the I-COACH system. Thus, by adjusting the AM-CPM, the I-COACH system can quickly achieve DOF imaging of samples with different thicknesses.

**Figure 8:**
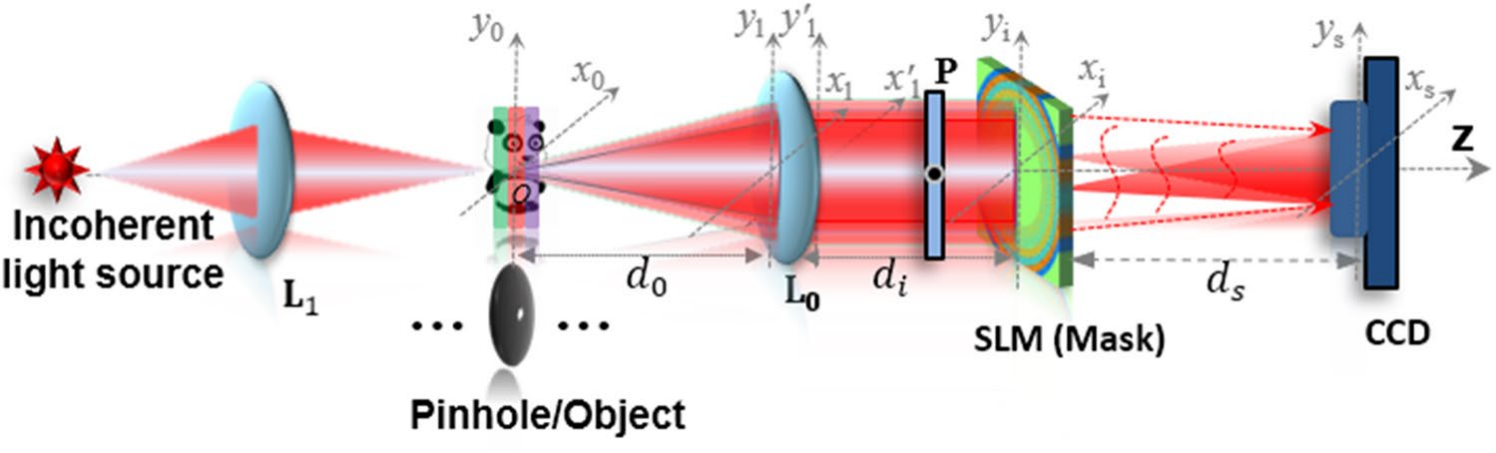
Optical setup of I-COACH system with DOF extension.

## Discussion

In this paper, we have proposed a design method for implementing a depth-of-field phase mask AM-CPM for the I-COACH system to further extend the imaging performance of the system. The source of axial defocus characteristics is demonstrated in I-COACH system and the defocus compensation function is determined, thus the AM-CPM with axial defocus aberration correction capability is proposed. The simulations and experimental results fully demonstrate the ability of the proposed method to extend the DOF by adjusting the parameters *m* and *α* to obtain different AM-CPM, thus enabling the I-COACH system to image samples at different depths rapidly and simultaneously. In conclusion, the proposed method improves the imaging performance of the I-COACH technology, and provides an important alternative method for further promoting the practical application in synthetic aperture imaging, scattered media imaging, microscopic imaging, and other fields.

## Methods

The optical setup of the I-COACH system with DOF extension and the coordinates of each plane are shown in Fig. 8. The object or pinhole is illuminated critically with incoherent light to guarantee complete incoherence between any two points on the object^10^. The diffracted light of the object is collected and collimated by the lens L_0_ with focal length *f*_L0_ located at a distance of *d*_*0*_ from the object. Then, the light is polarized by a polarizer **P** along the active axis of the SLM and totally modulated by a mask which is synthesized by a CPM, an annular multi-focal coded phase mask and a quadratic phase mask (QPM) with focal length *f*_slm_ loaded onto the SLM. The light modulated by the SLM is recorded by a charge coupled device (CCD) located at a distance of *d*_*s*_ from the SLM. In the general I-COACH setup, the light scattered from the mask is projected on the sensor plane by the QPM to satisfy the Fourier-transform relations between the CPM plane and the CCD plane, which means to *d*_*s*_ = − *f*_slm_ in the optical setup. Hence, the system is a regular lens imaging system when the SLM is only loading QPM, and an I-COACH system when a mixed mask is loaded.

When a point object is illuminated in the above optical setup, the light field of the point object on the SLM and CCD planes given as respectively,

1 $$U_{s} \left( {\overline{{r_{i} }} ;d_{0} } \right) = C_{1} \cdot Q\left( {\frac{{\overline{{r_{0} }} }}{{d_{0} }}} \right)Q\left( {\frac{{\overline{{r_{i} }} }}{{d_{i} }}} \right)\Im_{2D} \left\{ {A\left( {\overline{r} } \right)Q\left( {\frac{{d_{i} f_{{L_{0} }} + d_{0} f_{{L_{0} }} - d_{0} d_{i} }}{{d_{0} d_{i} f_{{L_{0} }} }}\overline{r} } \right)} \right\}{\kern 1pt} ,$$

2 $$U_{c} \left( {\overline{{r_{s} }} ;d_{s} } \right){\kern 1pt} {\kern 1pt} = {\kern 1pt} {\kern 1pt} {\kern 1pt} {\kern 1pt} C_{2} \cdot Q\left( {\frac{{\overline{r}_{s} }}{{d_{s} }}} \right)\Im_{2D} \left\{ {Q\left( {\frac{{d_{s} { + }f_{slm} }}{{d_{s} f_{slm} }}\overline{r}_{i} } \right){\kern 1pt} {\kern 1pt} U_{s} \left( {\overline{{r_{i} }} ;d_{0} } \right)\exp \left[ {i \cdot \phi (\overline{{r_{i} }} )} \right]} \right\},$$

where $\Im_{2D}$ is the 2D Fourier transform. $C_{1} = {{ - 1} \mathord{\left/ {\vphantom {{ - 1} {\lambda^{2} d_{i} d_{0} }}} \right. \kern-0pt} {\lambda^{2} d_{i} d_{0} }}$, $C_{2} = {1 \mathord{\left/ {\vphantom {1 {\lambda^{2} d_{s} }}} \right. \kern-0pt} {\lambda^{2} d_{s} }}$, $\overline{{r_{i} }} = \left( {x_{i} ,y_{i} } \right)$ and $\overline{{r_{s} }} = \left( {x_{s} ,y_{s} } \right)$ are transverse location vector on the SLM and CCD, respectively. $\overline{{r_{0} }} = \left( {x_{0} ,y_{0} } \right)$ and $\overline{r} = \left( {x^{^{\prime}} ,y^{^{\prime}} } \right)$ are transverse location vector on the object and rear plane of lens L_0_, respectively. Q is a quadratic phase function and given by $Q\left( {a\overline{r} } \right) = \exp \left[ {ia\pi \lambda^{{{ - }1}} \left( {x^{2} + y^{2} } \right)} \right]$. $A\left( {\overline{r} } \right)$ is the aperture function of the rear plane of the lens L_0_, where the value less than the lens L_0_ diameter *R* is 1 otherwise 0. *Φ* is a phase synthesized by a CPM, an AM-CPM and a QPM.

According to the above equations and optical setup, it can be seen the existence of quadratic function $Q\left( {\frac{{d_{i} f_{{L_{0} }} + d_{0} f_{{L_{0} }} - d_{0} d_{i} }}{{d_{0} d_{i} f_{{L_{0} }} }}\overline{r} } \right)$ which is defined as axial defocus aberrations make the relationship between the object and image plane not satisfy the conjugate relationship of a well-focused of the lens. Therefore, the distance *d*_*0*_ of point-object varies along **z**-axis will result in imaging defocus which should be compensated for extending imaging DOF. Unconventionally, we proposed a method of annular multi-focal coded phase mask that is multiplexed on the imaging system pupil plane to compensate simultaneously the various of defocus aberration. In our proposal, the axial defocus compensation function *ξ* is defined as,

3 $$\xi = \exp \left[ {j2\pi \lambda^{{{ - }1}} m\alpha \left( {x_{i}^{2} + y_{i}^{2} } \right)} \right],$$

where *m* and *α* are the designed parameters which can be setting respectively to compensate the different defocus. Let the conjugate relationship between axial defocus aberrations and axial defocus compensation function be fulfilled, and they satisfy the relationship of Eq. (4) at the same time,

4 $$m\alpha = \frac{{d_{0} d_{i} - d_{i} f_{{L_{0} }} - d_{0} f_{{L_{0} }} }}{{2d_{0} d_{i} f_{{L_{0} }} }}.$$

In Eq. (4), the parameters *d*_*i*_ and *f*_L0_ are determined according to the system, and *d*_*0*_ is related to the axial distribution depth of the 3D object, so that different axial locations *d*_*0*_ correspond to different *m* and *α*. In order to achieve continuous correction of axial defocus aberration within the depth of field *d*_*0*_, a space division multiplexing method is designed to divide the phase mask into annular equal-area zones. The ring zones with different focal lengths achieve the correction of the corresponding the different axial defocus aberrations. Thus, according to Eq. (4), the AM-CPM is designed as,

5a $$AM{ - }CPM{ = }\arg \left\{ {\exp \left[ {j2\pi \lambda^{{{ - }1}} \alpha f(\overline{{r_{i} }} )} \right]} \right\},$$

5b $$f(\overline{{r_{i} }} ) = \left\{ \begin{gathered} n_{1} {\kern 1pt} \gamma^{2} ,{\kern 1pt} {\kern 1pt} {\kern 1pt} {\kern 1pt} {\kern 1pt} {\kern 1pt} {\kern 1pt} {\kern 1pt} {\kern 1pt} {\kern 1pt} {\kern 1pt} {\kern 1pt} {\kern 1pt} {\kern 1pt} {\kern 1pt} {\kern 1pt} {\kern 1pt} {\kern 1pt} {\kern 1pt} {\kern 1pt} {\kern 1pt} {\kern 1pt} {\kern 1pt} {\kern 1pt} \frac{{n_{1} }}{m} \le \gamma^{2} {\kern 1pt} {\kern 1pt} {\kern 1pt} {\kern 1pt} {\kern 1pt} < \frac{{2n_{1} + 1}}{2m}{\kern 1pt} {\kern 1pt} \hfill \\ n_{2} {\kern 1pt} \gamma^{2} ,{\kern 1pt} {\kern 1pt} {\kern 1pt} {\kern 1pt} {\kern 1pt} {\kern 1pt} {\kern 1pt} {\kern 1pt} {\kern 1pt} {\kern 1pt} {\kern 1pt} {\kern 1pt} {\kern 1pt} {\kern 1pt} {\kern 1pt} {\kern 1pt} {\kern 1pt} {\kern 1pt} {\kern 1pt} {\kern 1pt} \frac{{ - 2n_{2} + 1}}{2m} \le \gamma^{2} {\kern 1pt} {\kern 1pt} {\kern 1pt} {\kern 1pt} {\kern 1pt} < \frac{{ - n_{2} { + }1}}{m} \hfill \\ \end{gathered} \right.,$$

where to facilitate the division of mask, the* m* is an integer and is also defined as the maximum number of rings of the AM-CPM. *n*_*1*_ = [0,1…*m*–1]and *n*_*2*_ = [− *m* + 1,− *m* + 2…0]. In Eq. (5b), *n*_*1*_ and *n*_*2*_ are realized for the defocus compensation of different axial distances on the left and right sides of the front focal plane of the lens L_0_, respectively. Thus, *n*_*1*_ and *n*_*2*_ are not larger than the absolute value of *m*. $\gamma { = }\sqrt {{{\left[ {\left( {m_{i} \Delta x} \right)^{2} + \left( {n_{i} \Delta y} \right)^{2} } \right]} \mathord{\left/ {\vphantom {{\left[ {\left( {m_{i} \Delta x} \right)^{2} + \left( {n_{i} \Delta y} \right)^{2} } \right]} {\left( {lx + ly} \right)}}} \right. \kern-0pt} {\left( {lx + ly} \right)}}}$,$\Delta x$ and $\Delta y$ are the sampling interval. *m*_*i*_ and *n*_*i*_ are coordinates in pixels. $lx = \left( {M\Delta x} \right)^{2}$ and $ly = \left( {N\Delta y} \right)^{2}$,M and N are the maximum number of pixels in the transversal and vertical coordinates. In other words, $f(\overline{{r_{i} }} )$ realizes the different ring zones with different focal lengths, and $\alpha f(\overline{{r_{i} }} )$ works together to realize further correction for different focal lengths with different ring zones. The adjacent annular zones of the AM-CPM have axisymmetric defocus aberration compensation functions to extend the DOF of the imaging system. Therefore, by modulating the parameters *m* and* α*, The AM-CPM with different DOF extension capabilities can be obtained.

## Data Availability

The datasets used and/or analyzed during the current study available from the corresponding author on reasonable request.
